# Preterm Preeclampsia and Timing of Delivery: A Systematic Literature Review

**DOI:** 10.1055/s-0038-1668529

**Published:** 2018-08

**Authors:** Tatiana Henriques Leite, Vitor Barreto Paravidino

**Affiliations:** 1 Institute of Social Medicine, Universidade do Estado do Rio de Janeiro, Rio de Janeiro, RJ, Brazil

Dear Editor,

Hypertensive disorder is the major cause of maternal mortality and morbidity in Brazil and Latin America.^1^ Immediate delivery improves maternal and perinatal outcomes in preeclampsia cases over 37 weeks of gestation.^2^ ^3^ However, there is no consensus in the literature regarding preeclampsia cases between 34 and 37 weeks of gestation. Guida et al (2017)^4^ conducted a systematic review to determine the best timing of delivery for women diagnosed with preeclampsia before 37 weeks of gestation, providing an update on the available evidence. This is an important issue. However, some limitations of this review should be discussed.

The most critical issue regards the search strategy. First, the authors used only one database (Medline). According to the Cochrane Handbook for Systematic Reviews, three databases should be considered when searching for trials: Medline, Embase and CENTRAL.^5^ In Brazil, the Ministry of Health recommends that a systematic review should include at least five databases; four essential databases (Medline, Embase, CENTRAL and Lilacs) and one area-specific database.^6^ This is an important issue because this procedure ensures that all evidence can be found. Secondly, the search strategy was limited to a 3-year period. This approach ignores all evidence produced before and is not recommended, especially in an update paper.^5^ Lastly, the authors reported that the search in the Medline was performed using Medical Subject Headings (MeSh) (*preeclampsia*, *parturition* and *timing of delivery*). However, these search terms are not sufficient, and MeSh such as “*Labor*, *Obstetric*,” “*Delivery*, *Obstetric*” and “*labor*, *Induced*” should also be included. All these limitations combined would result in missing articles, influencing the results of this review.

Another important limitation is the lack of risk of bias assessments (qualitative assessment). This tool evaluates the risk of overestimating or underestimating the true effect of the intervention. This is the only available strategy to evaluate internal validity—an important criterion in epidemiologic studies. Therefore, according to the Cochrane Handbook for Systematic Reviews, a qualitative assessment is part of the systematic review method.^5^

In addition, Guide et al (2017)^4^ pointed out several recommendations based on the results of this review. However, these recommendations were not classified according to the grading of recommendations assessment, development and evaluation (GRADE) system, taking into account the level of evidence and grading of recommendations.^7^

In summary, this review analyzed an important question; however, the authors should have performed an exhaustive search of the literature and used an appropriate methodological approach. Due to these limitations, any conclusion or recommendation concerning the results of this review should be interpreted with caution.
